# The TAM, a Translocation and Assembly Module for protein assembly and potential conduit for phospholipid transfer

**DOI:** 10.1038/s44319-024-00111-y

**Published:** 2024-03-11

**Authors:** Kwok Jian Goh, Christopher J Stubenrauch, Trevor Lithgow

**Affiliations:** 1 Centre to Impact AMR, Monash University, Melbourne, VIC 3800 Australia; 2 Infection Program, Biomedicine Discovery Institute and Department of Microbiology, Monash University, Melbourne, VIC 3800 Australia

**Keywords:** Outer Membrane Biogenesis, Beta-barrel Proteins, BAM Complex, Lipid Transport, TamB, Membranes & Trafficking, Microbiology, Virology & Host Pathogen Interaction

## Abstract

The assembly of β-barrel proteins into the bacterial outer membrane is an essential process enabling the colonization of new environmental niches. The TAM was discovered as a module of the β-barrel protein assembly machinery; it is a heterodimeric complex composed of an outer membrane protein (TamA) bound to an inner membrane protein (TamB). The TAM spans the periplasm, providing a scaffold through the peptidoglycan layer and catalyzing the translocation and assembly of β-barrel proteins into the outer membrane. Recently, studies on another membrane protein (YhdP) have suggested that TamB might play a role in phospholipid transport to the outer membrane. Here we review and re-evaluate the literature covering the experimental studies on the TAM over the past decade, to reconcile what appear to be conflicting claims on the function of the TAM.

## Abstract (web summary)

This review discusses evidence for the function of the Translocation and Assembly Module (the TAM) in outer membrane β-barrel protein assembly and assesses whether the TAM could potentially have a dual function in the phospholipid equilibration between the inner and outer membranes.

## Graphical abstract

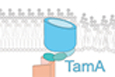

## Introduction

Across the great diversity of Gram-negative bacteria, the most characteristic common feature is the presence of two membranes: an inner membrane that provides for containment of the cytoplasm, and an outer membrane that provides a second layer of protection to the underlying bacterial cell. The presence of an outer membrane adds complexity to processes needed for cell growth: since the lipid and protein components of the outer membrane are all synthesized in the inner compartments of the cell, translocation across the inner membrane and across the periplasmic compartment to the outer membrane is essential. These protein and lipid transport processes are collectively referred to as outer membrane biogenesis. Phospholipids and lipopolysaccharides are transferred from their site of synthesis in the inner membrane to populate the outer membrane, and the nascent forms of membrane proteins are delivered from the cytoplasmic ribosomes to translocate across the inner membrane and periplasm, for assembly into the outer membrane. Currently, as the field of bacterial cell biology matures into one that can address processes such as outer membrane biogenesis at a systems level, hugely interesting questions are arising. A long-standing mystery in outer membrane biogenesis has been how phospholipids flow to the outer membrane (Powers and Trent, 2019; Yeow and Chng, 2022). One model for the process proposes that this transport is by diffusive flow, allowing for phospholipid species to equilibrate via channels that would connect the inner and outer membranes (Grimm et al, 2020; Yeow and Chng, 2022). In synthetic genetic screens addressing factors that assist the main phospholipid channel YhdP to equilibrate phospholipids into the outer membrane (McDonnell et al, 2023; Ruiz et al, 2021), the gene encoding TamB was identified, and questions are now being asked of whether TamB plays a role in phospholipid equilibration instead of β-barrel protein assembly (Kumar and Ruiz, 2023). In this review of the literature, we highlight that the Translocation and Assembly Module (the TAM) does function in β-barrel protein assembly for many proteins of the outer membrane and assess whether it could potentially have a dual function in also serving as an important redundancy in the phospholipid equilibration process between the inner and outer membranes.

## One component of the TAM is an Omp85 superfamily protein

**Figure 1:**
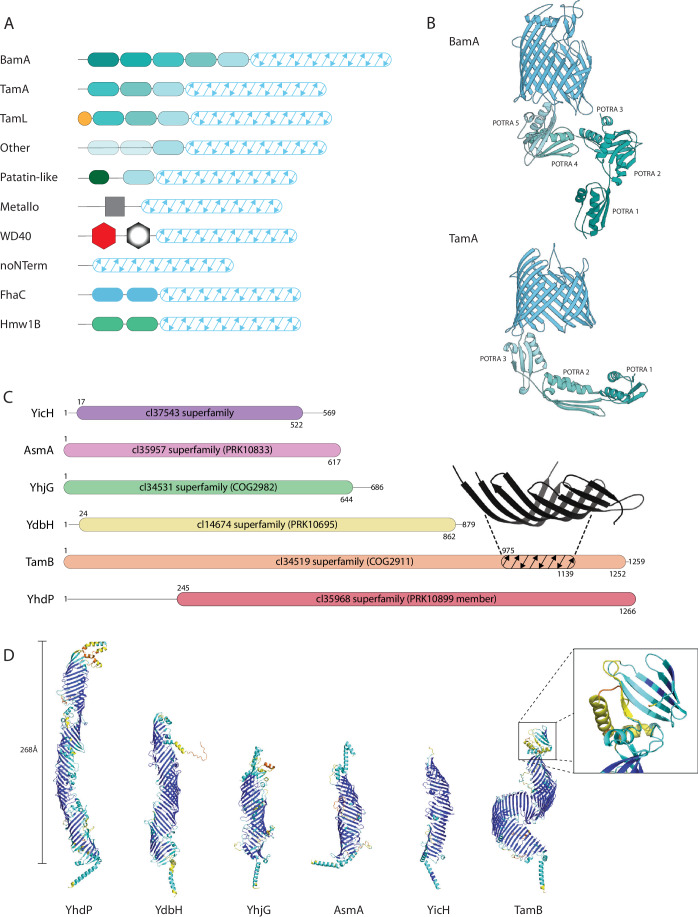
Domain architectures within the Omp85 and AsmA superfamilies of proteins. (**A**) Diagrammatic representation of the domain structure of BamA, TamA, and the other eight Omp85 protein families found in bacteria (Heinz and Lithgow, 2014). N-terminal POTRA domains are shown in shades of green/blue, and the alternative N-terminal domains are shown in red, gray, or black as previously detailed (Heinz and Lithgow, 2014), and the β-barrel surface antigen domain is shaded with diagonal arrows. (**B**) The crystal structure of TamA (PDB: 4C00) (Gruss et al, 2013) and BamA (PDB: 5D0Q) (Noinaj et al, 2013) are shown portraying the arrangement of the POTRA domains with respect to the membrane-embedded β-barrel domain. (**C**) Diagrammatic representation of the domain structure of TamB and related AsmA-superfamily proteins are indicated, with the residue numbers shown at the C-terminus of each protein, the domains determined by CD-search (https://www.ncbi.nlm.nih.gov/Structure/cdd/wrpsb.cgi) are drawn to scale. (inset) The crystal structure of the β-taco fragment of TamB (PDB: 5VTG) (Josts et al, 2017), colored black, is shown diagrammatically in TamB as residues 975–1139. (**D**) Structure predictions for the *E. coli* representatives of the YhdP family, YdbH family, YhjG family, AsmA family YicH family and TamB family of proteins using Alphafold (Jumper et al, 2021). The predictions made use of default parameters and the color-coding is standard where dark blue represents the highest-confidence prediction and red-yellow denotes predictions of less confidence. In a similar analysis, the length projection of the periplasmic domain of YhdP was calculated to be 268 Å (Kumar and Ruiz, 2023). (Inset) The Alphafold prediction of the pseudosubstrate domain of TamB that mediates interaction with the N-terminal most POTRA domain of TamA, as classified by sequence features (Heinz et al, 2015) and biochemical analysis (Selkrig et al, 2015; Selkrig et al, 2012).

With their first description in bacteria twenty years ago (Voulhoux and Tommassen, 2004) there was debate about whether Omp85 proteins were involved in β-barrel protein assembly (Voulhoux et al, 2003) or lipid transport between the inner and outer membranes (Genevrois et al, 2003). Omp85 proteins have been characterized by a conserved bacterial surface antigen domain (Fig. 1A), and the associated N-terminal domains called “POTRA” (for polypeptide transport-associated motifs) that bind partner proteins and protrude into the periplasm (Doyle and Bernstein, 2022; Gruss et al, 2013; Simmerman et al, 2014; Webb et al, 2012). The core of the β-barrel protein assembly machinery, BamA, is a member of the Omp85 superfamily of proteins (Doyle and Bernstein, 2022; Noinaj et al, 2017; Ranava et al, 2018; Ricci and Silhavy, 2019). Genome sequence analysis in *E. coli* revealed a second member of the Omp85 family of proteins (Stegmeier et al, 2007), which was designated TamA (Selkrig et al, 2012) and suggested to contribute to the β-barrel protein assembly machinery. The protein was encoded in an operon, where the *ytfM* gene encoded the Omp85 protein TamA and the *ytfN* gene encoded the protein TamB. Antibodies raised to the proteins showed them to be present in distinct subcellular locations: TamA in the outer membrane and TamB in the inner membrane (Selkrig et al, 2012). Co-immunoprecipitation assays and blue native-PAGE analysis revealed that TamA forms a 1:1 complex with TamB (Selkrig et al, 2015; Selkrig et al, 2012; Shen et al, 2014).

A comprehensive analysis of bacterial genomes to detect and classify all members of the Omp85 superfamily of proteins revealed ten distinct protein families within the superfamily (Heinz and Lithgow, 2014). BamA is one family, TamA is a second family, and there are eight further groups (Fig. 1A). Some of these have functions that are established to be unrelated to β-barrel protein assembly. For example, two of these families represent the two distinct transporters, FhaC and Hmw1B, categorized as TypeVb secretion systems (Guerin et al, 2017) with others predicted to have distinct functions such as a family of metalloproteases and the patatin-type virulence proteins (Hanson et al, 2023). Across the ten families of Omp85 proteins (Fig. 1A), seven of the families have POTRA domains and the POTRA domains within each family have distinguishing sequence characteristics (Heinz and Lithgow, 2014). These characteristics provide for distinct interactions with partner proteins, as illustrated in crystal structures and cryo-EM structures of the β-barrel assembly machinery (BAM) complex (Bakelar et al, 2016; Gu et al, 2016; Han et al, 2016; Iadanza et al, 2020; Jansen et al, 2015; Wu et al, 2021), showing the POTRA domains of BamA (Fig. 1B) provide specific binding sites for the lipoprotein subunits BamB and BamD (Fig. 1A). Likewise, for TamA the POTRA domains (Fig. 1B) are necessary and sufficient for interaction with TamB (Bamert et al, 2017; Selkrig et al, 2012). NMR analysis has shown that the first POTRA domain of TamA is specialized in shape and positively charge surface features, with BN-PAGE and further experiments suggesting that this first POTRA domain binds the partner protein TamB (Selkrig et al, 2015).

## One component of the TAM is an AsmA-superfamily protein

Just as TamA is a member of a protein superfamily, a comprehensive sequence analysis of bacterial genomes showed that TamB is a member of a protein superfamily (Douglass et al, 2022; Heinz et al, 2015; Ruiz et al, 2021), which shares similarities to the defining features of the repeating beta-groove superfamily (Neuman et al, 2022). In terms of the bacterial superfamily of proteins, the *E. coli* protein AsmA is used as the descriptive term: hence AsmA-like proteins (Heinz et al, 2015; Ruiz et al, 2021) and AsmA-like protein family (Douglass et al, 2022). Hidden Markov Model analysis revealed the relationship of the TamB protein family to other protein families in this superfamily (Fig. 1C), where a cluster distribution analysis highlighted two important considerations: (i) that while TamB can be found in all bacterial Phyla with an outer membrane, the other proteins (AsmA, YicH, YhjG, etc.) have more limited occurrences across bacterial Phyla and, (ii) that while the protein clusters show sequence-based similarity between the groups, the protein groups are discretely clustered in a way that would suggest structural and possible functional differences (Heinz et al, 2015). In this regard, the AsmA-like proteins are perhaps analogous to the Omp85 superfamily of proteins, where sequence-based and broad structural similarities do not necessarily dictate wholly conserved functions (Doyle and Bernstein, 2022; Grass et al, 2015; Hanson et al, 2023; Nash and Cotter, 2019).

Re-evaluation of the *E. coli* protein sequences in terms of conservation in their domains with the web-based conserved domain search tool CD-search (Geer et al, 2002) documents those differences in each cluster of the AsmA-superfamily (Fig. 1C). Conversely, and importantly, the use of the structural predictor AlphaFold highlights the overall conservation in structure, as seen for the six *E. coli* proteins: AsmA, YicH, YhjG, YhdP, YdbH, and TamB (Fig. 1D). Tools like CD-search make use of position-specific conservation information, and thereby discriminate groups of proteins based on how closely related they are in overall terms. Thus, while each of these proteins carries domains that can be collectively referred to as repeating beta-groove structures (Neuman et al, 2022), the sensitivity of CD-search detection reveals that these are not equivalent with only AsmA, YicH, YhjG conforming strictly to the AsmA domain, YhdP conforming to the extended (AsmA2) version of the AsmA domain, and YdbH and TamB being conserved domains distinct from the others (Fig. 1C,D). The differences in sequence conservation revealed in the HMMR analysis and CD-search predictions may reflect important differences, such as the potential for super-coiling and predicted flexibility previously ascribed to TamB (McDonnell et al, 2023; Ruiz et al, 2021; Shen et al, 2014), as well as the prospective pseudosubstrate domain (Heinz et al, 2015) formed from the final six β-strands at the C-terminal end of TamB (Fig. 1D, inset), with this feature interacting with TamA (Selkrig et al, 2015; Selkrig et al, 2012). Consistent with the TamB-TamA interaction, comparative genomic analysis has revealed that most, but not all, bacterial lineages that encode a TamB, also encode a TamA (Heinz et al, 2015; Stubenrauch et al, 2022). In species such as *Borrelia burgdorferi*, a bacterium where TamB is present but TamA is not, co-immunoprecipitation analysis suggests that TamB is a partner protein of BamA (Iqbal et al, 2016), contributing its function to β-barrel protein assembly through the BAM complex (Heinz et al, 2015; Iqbal et al, 2016; Stubenrauch et al, 2016b).

Despite a decade of work in several labs, the structure of TamB has remained elusive (Box 1). Only a section of the beta-groove has been crystallized and the structure of this β-taco revealed a taco-shaped structure with a hydrophobic groove running the length of this fragment (Josts et al, 2017). The advent of AlphaFold (Jumper et al, 2021) provided a means around this impasse and the structural model for the whole TamB protein (Fig. 1D) predicts: (i) that the C-terminal pseudosubstrate domain (Heinz et al, 2015) forms a sector of an amphipathic β-barrel, akin to the substrates assembled by the TAM (Fig. 1D, inset), and (ii) the structural model of the entire repeating beta-groove domain that is consistent with the features of the β-taco crystal structure.

**Figure 2:**
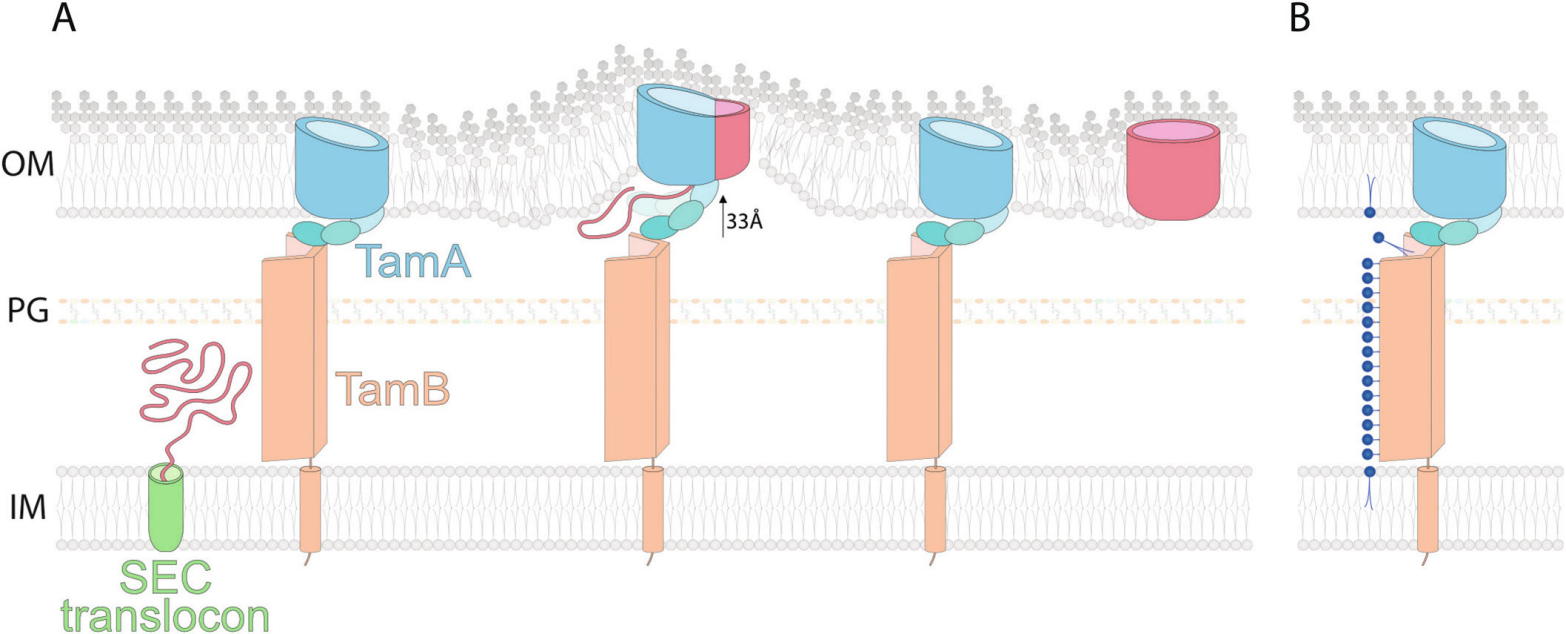
Models for the function of TamB. (**A**) A model for TAM function drawing on measurements made by (Shen et al, 2014) and (Selkrig et al, 2015) to suggest dynamics in the movement of the TAM during its reaction cycle to assemble β-barrel proteins (red) after their translocation across the inner membrane (IM) is mediated by the SEC translocon. Measurement of the distance across the periplasm varies with an average that can be considered ~260 Å, with the peptidoglycan layer (PG) situated ~100 Å from the outer membrane (OM) (Asmar et al, 2017; Cohen et al, 2017; Mandela et al, 2022) and this scale is approximated in the diagram. The graphical representation of the topology of the TAM subunits, TamA and TamB, is based on (i) the extended TamB conformation (McDonnell et al, 2023) of 246 Å which would be necessary and sufficient to reach and contact TamA, and (ii) the crystal structure of TamA that has the POTRA domains approximately 44 Å from the outer membrane surface. Measurements by neutron reflectometry showed the POTRA domain is able to move to at least 77 Å from the membrane when it encounters its substrate (Shen et al, 2014). This movement of +33 Å would either locally deform the outer membrane (shown) or require a tilting of TamB to accommodate the downward movement. (**B**) A model for TAM function by analogy to the function of YhdP, where phospholipids have been cross-linked to the hydrophobic groove in YhdP (Cooper et al, 2023). By analogy, the hydrophobic groove in TamB might provide for equilibration of phospholipids between the inner membrane and outer membrane.

Viewing the AlphaFold prediction in three dimensions also suggests that the hydrophobic groove seen in the β-taco runs the length of the β-structured domain of TamB (Rai et al, 2023), the definition suggested for the repeating beta-groove superfamily (Neuman et al, 2022). The simple AlphaFold model for TamB suggests a supercoil in the β-structured domain to generate a protein that would be 17.8 nm (i.e., 178 Å) in length, insufficient to reach the TamA partner protein. However, modeling with molecular dynamics supports an extension of the supercoil that would stretch the TamB to be 24.6 nm (i.e., 246 Å) in length (McDonnell et al, 2023), this being sufficient to penetrate the peptidoglycan layer and reach TamA (Fig. 2A). AlphaFold is not trained to take account of subcellular context (Zhu et al, 2023), so the need to interact with the TamA partner protein in the outer membrane as well as pass through the peptide-environment of the peptidoglycan layer, may add further to the stability of the TAM in the context of a native cell envelope. A structure of the intact TamA-TamB complex is needed in order to resolve the question of how it spans across the periplasm (Box 1).

Box 1 In need of answers

- Structural information on TamB to assess the reliability of the AlphaFold predicted structure, particularly in terms of the predicted shape that would limit TamB penetration through the peptidoglycan layer in situ.
- Structural assessment, for example, using cryo-electron tomography, to refine the structure of the intact TAM (i.e., TamA + TamB) in situ.
- Biochemical assessment of the involvement and capacity of TamB and YdbH as ‘back-up’ phospholipid conduits in *E. coli* cells lacking a functional YhdP.
- Quantitative measurements of phospholipid flux and equilibration, as has been done for protein transport, to determine the relative impact of each component mediating phospholipid transport.

## Evidence that the TAM functions in outer membrane protein assembly

The protein names TamA and TamB and the machine that they form, the TAM, refers to a Translocation and Assembly Module, which was functionally defined as a module of the β-barrel assembly machinery. The first indications that the TAM functioned in membrane protein assembly focussed on three autotransporters: p1121 from *Citrobacter rodentium* and the *E. coli* proteins EhaA and Ag43 (Selkrig et al, 2012). The complicated topology of autotransporters consists of a large β-helical “passenger” domain that threads through their C-terminal β-barrel domain to be surface-exposed (Albenne and Ieva, 2017). Loss of TamA in *C. rodentium* led to the absence of the major outer membrane protein p1121 from purified outer membranes, and in a recombinant system whereby *E. coli* was overexpressing p1121, protease shaving analysis revealed a severe drop in the p1121 surface-exposed passenger domain when either TamA or TamB were absent. Functional analysis of EhaA or Ag43, which cause wild-type cells to aggregate in solution, revealed that in Δ*tamAB* double mutants, *E. coli* cells could no longer aggregate and instead remained in the suspension. Subcellular fractionation showed that in the absence of TamA, Ag43 was still localized to the outer membrane, but protease shaving experiments revealed that the passenger domain was not properly surface-exposed (Selkrig et al, 2012). The conclusion made in this study was that, in the absence of the TAM, the β-barrel assembly machinery was overwhelmed by the heterologous expression of these substrates. Whether this represents a pure quantitative overload of protein substrate or additional factors are at play remains to be determined.

Multiple direct measurements in *E. coli* have since established that the function of the TAM contributes toward catalyzing the assembly of several β-barrel protein types of complicated topology into the bacterial outer membrane (Table 1). Experimental data has been presented showing the increased efficiency that the TAM brings to the assembly of autotransporter adhesins (Selkrig et al, 2012), inverse autotransporter adhesins, such as FdeC and Intimin (Heinz et al, 2016), and usher proteins (Heinz et al, 2016; Stubenrauch et al, 2016a; Stubenrauch et al, 2017). To directly measure the activity of the TAM in the process, three assay systems have been established to quantify the TAM catalyzing the assembly of means β-barrel proteins.

**Table 1:** Experimental evidence for diverse outer membrane precursor substrates of the TAM.

Substrate	Method of detection	Reference
β-strand peptide	Direct TamA-peptide interaction	Bamert et al, 2017
p1121 (autotransporter)	Protease shaving: assembly defect in Δ*tamA* or Δ*tamB* cells	Selkrig et al, 2012
EhaA (autotransporter)	Aggregation assay: assembly defect in Δ*tamAB* cells	Selkrig et al, 2012
Ag43 (autotransporter)	Aggregation assay: assembly defect in Δ*tamAB* cells; restored on complementation with TamAB Protease shaving: assembly defect in Δ*tamA* cells	Selkrig et al, 2012
Ag43 (autotransporter)	Magnetic contrast neutron reflectometry: Ag43 assembly dependent on TamA or TamAB, with structural changes in TamA measured upon Ag43 folding	Shen et al, 2014
Ag43 (autotransporter)	Quartz crystal microbalance: Ag43 assembly dependent on TamA or TamAB; Ag43 folding strongly inhibited with deletion of TamA POTRA 1, and abolished when POTRA 1–2 or POTRA 1-3 deleted	Selkrig et al, 2015; Shen et al, 2014
Intimin (adhesin)	[^35^S] Pulse-chase labeling: assembly defect in Δ*tamA* cells; Assembly restored on complementation with TamA	Heinz et al, 2016
FdeC (adhesin)	[^35^S] Pulse-chase labeling: assembly defect in Δ*tamA* cells	Heinz et al, 2016
PapC (usher)	[^35^S] Pulse-chase labeling: assembly defect in Δ*tamA* cells	Stubenrauch et al, 2016a
HtrE (usher)	[^35^S] Pulse-chase labeling: assembly defect in Δ*tamA* cells	Stubenrauch et al, 2016a
YbgQ (usher)	[^35^S] Pulse-chase labeling: assembly defect in Δ*tamA* cells	Stubenrauch et al, 2016a
YfcU (usher)	[^35^S] Pulse-chase labeling: assembly defect in Δ*tamA* cells	Stubenrauch et al, 2016a
UshC (usher)	[^35^S] Pulse-chase labeling and assembly kinetics: Assembly defect in Δ*tamA* or ΔtamB cells; assembly restored on complementation with TamA or TamB, respectively	Stubenrauch et al, 2017
TolC (efflux pump)	Direct TamA-TolC interaction by cross-linking	Stubenrauch et al, 2022
FimD (usher)	[^35^S] Pulse-chase labeling and transport kinetics: assembly defect in Δ*tamA* or Δ*tamB* cells;	Bamert et al, 2017; Josts et al, 2017; Stubenrauch et al, 2016a; Stubenrauch et al, 2022
Phospholipids	Synthetic genetic growth and antimicrobial sensitivity defects in Δ*yhdP*Δ*tamB* or Δ*yhdP*Δ*tamA* cells	Douglass et al, 2022; Rai et al, 2023; Ruiz et al, 2021
Phospholipids	Steady-state lipid analysis: Δ*tamB*,Δ*ydbH* mutants with reduced Δ*yhdP* has reduced phospholipid content in outer membrane	Douglass et al, 2022
Phospholipids	Δ*yhdP*,Δ*tamB* cells have synthetic SDS-sensitivity	Ruiz et al, 2021

In one assay system, purified TamA reconstituted into a membrane layer on a gold chip permitted measurements of protein movement by neutron reflectometry (Shen et al, 2014) and by quartz crystal microbalance measurements (Selkrig et al, 2015). TamB was added to the system and neutron reflectometry showed TamB bound TamA via the POTRA domain that extended 4.4 nm from the membrane surface. Addition of a purified, urea-denatured OMP to the system resulted in the movement of the POTRA domains: moving from 44 Å (i.e., 4.4 nm) to be 77 Å (7.7 nm) outwards from the membrane. The nett result of the movement of the TAM was to deliver the β-barrel protein into the membrane layer. TamB was not required for this activity: neither for the movement of the POTRA domain of TamA, nor the insertion of the substrate into the membrane. The study demonstrated that the purified TAM reconstituted into a membrane is necessary and sufficient to insert a β-barrel protein into the membrane, and that TamB is not released from TamA in order for the functional dynamics of the system (Shen et al, 2014). Using the reconstituted TamA on a quartz crystal microbalance provides for highly accurate and time-resolved monitoring of mass increases, and shows that TamB binds to TamA, but not to TamA lacking the first POTRA domain. This assay system was also used to show that while substrate binding by TamA does not require TamB, it is accelerated by the presence of TamB (Selkrig et al, 2015).

The second assay system directly monitors the time course for assembly of a β-barrel protein and provided the details for the assembly pathway (Fig. 2A). Selective labeling with ^35^S-amino acids in vivo means β-barrel protein assembly is measured in intact cells (Heinz et al, 2016; Stubenrauch et al, 2016a; Stubenrauch et al, 2017). The assay system has been applied to show the function of the TAM in promoting efficient assembly of the fimbrial ushers PapC, HtrE, YbgQ, YfcU, UshC and FimD, and the inverse autotransporters, FdeC and Intimin (Table 1). For example, the fimbrial usher protein FimD needs both TamA and TamB for efficient assembly into the outer membrane, and the assay showed that their role is profound: with the TAM present, FimD assembly proceeds from the C-terminus and is complete in under 2 min. While the BAM complex can fold FimD in mutants without TAM, BAM-mediated assembly starts from an internal strand in FimD and the assembly of FimD takes 240 min (Stubenrauch et al, 2016a). Because the assay system can distinguish the folding of the N-terminal portion of the β-barrel from the C-terminal portion, this study provided the first experimental demonstration of the polarity of the mechanism for strand-wise β-barrel assembly (Stubenrauch et al, 2017). The phenotypic consequence of Δ*tamA* mutants and Δ*tamB* mutants showed that they extend fimbriae three times slower than that of wild-type *E. coli*; this process is not essential to viability in the lab, but the rate of fimbrial deployment may have consequences in the environment.

A third assay system uses disulfide-mediated cross-linking (Kuszak et al, 2015) to address whether the TAM participates in folding of TolC, a β-barrel protein where the BAM complex provides for efficient folding (Stubenrauch et al, 2016a; Werner and Misra, 2005). Loss of either TamA or TamB, did not significantly affect the rate of assembly of ^35^S-TolC (Stubenrauch et al, 2016a), and there was no functional defect in antibiotic efflux either (Stubenrauch et al, 2022). To test the hypothesis that nascent TolC substrate molecules engage the lateral gate (Bamert et al, 2017) in TamA, a series of cysteine residues were incorporated into the gate and used to assess substrate engagement via disulphide cross-linking experiments. TolC engages the lateral gate of TamA in transit into the bacterial outer membrane, contributing to a process where it is the BAM complex activity that is rate-limiting (Stubenrauch et al, 2022).

## Does the TAM also assist in phospholipid equilibration?

A genetic screen for suppressors of a dominant *mlaA* mutant discovered that YhdP functions in phospholipid flow to the outer membrane (Grimm et al, 2020). However, it was noted that *E. coli* also encodes five other AsmA-like proteins: YdbH, AsmA, YicH, YhjG, and TamB (Fig. 1C), and sequence analysis showed that these five proteins form highly distinct protein clusters across various bacterial phyla (Heinz et al, 2015). Recent studies are addressing whether some or all these five proteins have structures that could provide a hydrophobic groove for lipid equilibration between the two membranes in *E. coli*, particularly when YhdP function is compromised (McDonnell et al, 2023; Rai et al, 2023; Ruiz et al, 2021). The structures on which these suggestions are made are predicted by Alphafold (Fig. 1D).

YhdP provides the primary conduit for phospholipid flow, with a suggested capacity for high-flux transport (Grimm et al, 2020). This prediction has been supported experimentally through molecular dynamics and structure-based analyses that show an uncharged, hydrophobic groove that could shield the acyl tails of phospholipid molecules traversing through the periplasmic space (Cooper et al, 2023). In the same study, in situ chemical cross-linking experiments confirmed the presence of phospholipids in this hydrophobic groove in YhdP that runs from the inner membrane to the outer membrane. To explore whether TamB might provide a redundancy for this phospholipid equilibration process, synthetic genetic interactions have been probed using growth phenotypes as readouts for lipid equilibration (Table 1). Growth phenotypes of deletion mutants made in a Δ*yhdP* background suggested that the other two largest members of the AsmA-protein superfamily (YdbH and TamB) provide the most cover for lipid equilibration in the absence of YhdP, and that deletion of all three genes generates a lethal phenotype in *E. coli* (Douglass et al, 2022; Ruiz et al, 2021). For example, Δ*yhdP*,Δ*tamB* cells have defects in outer membrane permeability as judged by reduced growth in the presence of SDS or antibiotics (Douglass et al, 2022; Ruiz et al, 2021) and Δ*ydbH*,Δ*tamB* cells with down-regulated YhdP have a decreased phospholipid content in their outer membranes (Douglass et al, 2022; Ruiz et al, 2021).

Taken together, the various recent studies suggest there are multiple passive channels for phospholipid equilibration across the *E. coli* cell envelope, with YhdP serving as the most capable conduit since it spans the inner and outer membranes and cells lacking YhdP are the most compromised in terms of growth and membrane phenotypes (Cooper et al, 2023; Douglass et al, 2022; Grimm et al, 2020; Rai et al, 2023; Ruiz et al, 2021). TamB (Fig. 2B) and YdbH appear to be bystanders, serving in phospholipid equilibration when YhdP is compromised. The extent to which the other three proteins (AsmA, YicH and YhjG) can contribute to the process of phospholipid flow to the outer membrane remains less clear (Box 1).

## With a BAM complex, who needs a TAM?

The BAM complex is essential for cell viability and undertakes the heaviest workload for membrane protein assembly. Recent analyses show it to be directly involved in the assembly of the major porins in *E. coli*, OmpC, and OmpF (Gunasinghe et al, 2018; Hussain et al, 2021; Thewasano et al, 2023). Many bacterial species like *E. coli* have an outer membrane characterized by a major porin, a highly-abundant integral membrane protein with a β-barrel structure that creates a central luminal pore large enough for nutrients and water-soluble drugs to enter the periplasm (Fairman et al, 2011; Koebnik et al, 2000; Prajapati et al, 2021). Quantitative analysis suggests that there could be as many as 34,500 molecules of major porin on the surface of each *E. coli* cell (Benn et al, 2021; Lithgow et al, 2023). In order to achieve and maintain this density of porins in the membrane through cycles of cell growth and division, the assembly rate has been calculated at ~860 molecules per minute assembled into the outer membrane (Lithgow et al, 2023). These calculations are in keeping with measurements of β-barrel assembly in intact *E. coli* cell experiments (Jansen et al, 2000; Reid et al, 1988; Smit and Nikaido, 1978; Stubenrauch et al, 2016a; Ursell et al, 2012) and this rate of substrate protein flux means that at any given time most of the active BAM complexes in each cell would be engaged in assembling a major porin. When expression of the gene encoding BamA is shut-down (Dunstan et al, 2015; Werner and Misra, 2005) it takes 3 h until cell division and other processes (Merdanovic et al, 2011; Werner and Misra, 2005) leave the *E. coli* population without measurable amounts of BamA and, at that time, the membranes demonstrate diminishing levels of OmpC (Dunstan et al, 2015).

Given the BAM complex is so well geared for major porin assembly, and sufficient to maintain cell viability, it seemed paradoxical that the *tamA-tamB* genes are a conserved feature in so many bacterial lineages (Heinz et al, 2015; Stubenrauch et al, 2022; Webb et al, 2012). What is the basis on which these genes are so strongly selected? Our hypothesis is that *tamA* and *tamB* genes contribute to membrane biogenesis outcomes that are not necessary for growth in resource-rich lab monocultures, namely for growth under conditions where BAM complex activity is suboptimal or to assemble complicated protein topologies including those recently acquired through lateral gene transfer. There is evidence consistent with this model: in the absence of the TAM, mutants constructed in several species show severe colonization and virulence defects: in species of *Salmonella* (Selkrig et al, 2012), *Brucella* (Bialer et al, 2019), *Klebsiella* (Jung et al, 2021; Struve et al, 2003), *Citrobacter* (Kelly et al, 2006; Selkrig et al, 2012), *Proteus* (Burall et al, 2004), *Aggregatibacter* (Gallant et al, 2008; Smith et al, 2016), *Edwardsiella* (Li et al, 2020) and *Vibrio* (Brooks et al, 2014). For example, studies with *Klebsiella pneumoniae* infection models showed that Δ*tamA* mutants are compromised in colonizing the mouse intestinal environment and are more readily cleared from lung infection sites and bloodstream infections by host immune defences (Jung et al, 2021). Thus, while not essential for growth in laboratory monocultures, the function of the TAM does provide a selective advantage under numerous environmental scenarios for various bacterial species.

Further support for this model comes from studies focussed on the usher proteins found in *E. coli*. The TAM is required for the efficient assembly of FimD, PapC, HtrE, YbgQ, YfcU and UshC (Stubenrauch et al, 2016a; Stubenrauch et al, 2017). FimD is the usher extruding Type 1 fimbriae of uropathogenic *E. coli* to colonize the epithelial surface of the bladder (Wu et al, 1996), PapC is the usher required for the production of the P fimbriae that mediate UPEC adherence in the upper urinary tract (Kuehn et al, 1992), HtrE is the usher for the assembly of Yad fimbriae (Korea et al, 2011), UshC is an usher with a discrete distribution in specific pathovars of extraintestinal pathogenic *E. coli* (ExPEC) and appears to have evolved from an usher found in *Enterobacter* spp. (Stubenrauch et al, 2017), while the ushers YbgQ and YfcU are conserved only in highly virulent *E. coli* pathotypes (Korea et al, 2010; Wurpel et al, 2013). While failing to extrude any of these fimbriae does not slow cell growth or prevent viability in the lab, it is a highly selectable feature in natural environments that could contribute to the selection pressure that ensured *tamA* and *tamB* are so highly conserved (Busch and Waksman, 2012; Heinz et al, 2015; Stubenrauch et al, 2022; Webb et al, 2012).

## Efficient assembly of foreign (alien) outer membrane proteins

Also not required under laboratory conditions, lateral gene transfer has had a huge impact in shaping the colonization of new environmental niches as evident in studies mapping the pangenome of *E. coli* (Fang et al, 2020; Yu et al, 2021). Compared to the core genome of the lab strain *E. coli* K-12 substr. MG1655, there are 60% of protein-coding genes in the enterohaemorrhagic *E. coli* O157:H7 and uropathogenic *E. coli* CFT073 that were acquired relatively recently via later gene transfer (Welch et al, 2002). As a result, bacterial populations sample genes that can shape the fitness and improve the competitiveness of clones to colonize new niches (Leimbach et al, 2013). However, to provide a phenotypic advantage, each newly acquired gene needs to be expressed and the proteins that they encode need to be assembled into a functional form. Failure to do so prevents the new phenotype from being advantageous and also presents potential risk to the cell including the inhibition of protein assembly rates, due to so-called “stuck intermediates” that accumulate from inefficient protein folding (Dorman, 2004; Kudla et al, 2009; Michael, 2017; Park and Zhang, 2012; Stubenrauch et al, 2017).

Tracing the distribution and evolution of the fimbrial usher UshC showed it to be a recent acquisition into a discrete group of *E. coli*, particularly a set of lineages of ExPEC (Stubenrauch et al, 2017). Experiments done in *E. coli* showed that the assembly efficiency of UshC is rate-limited by the TAM. Furthermore, it was demonstrated that if the predicted ancestral versions of the protein (from *Yersinia enterobacter* or *Enterobacter asburiae*) were transformed into *E. coli* to mimic a lateral gene transfer event, successful expression depended on the TAM in order that the *E. coli* host could assemble the foreign ushers into the outer membrane (Stubenrauch et al, 2017).

## Concluding remarks

TamB has been characterized as an essential subunit for the assembly of β-barrel proteins by the TAM. The periplasmic-exposed domain of TamB has a hydrophobic internal surface. Such a feature is found in many protein-binding factors, perhaps best known in the internal surface of the chaperone GroEL (Hayer-Hartl et al, 2016). In TamB, the hydrophobic surface has also been suggested to provide for phospholipid equilibration, given that the N-terminal signal anchor of TamB places it in the inner membrane (where phospholipids are made) and the C-terminal end of TamB contacts the outer membrane protein TamA. Before accepting that TamB contributes directly to phospholipid equilibration as well as β-barrel protein assembly, work would be needed to demonstrate the presence of a rivulet of phospholipids on TamB—as seen for YhdP (Cooper et al, 2023)—as the means for equilibrating lipids from the inner to outer membrane and establish how the phospholipids could travel across the POTRA domains of TamA to get into the outer membrane (Box 1).

The importance of biochemical demonstration of function is underscored by the analogous case of BamA. The breakthrough papers on BamA described it as functionating in β-barrel protein assembly into the outer membrane (Voulhoux et al, 2003) or in lipid transport between the inner and outer membranes (Genevrois et al, 2003). Upon understanding that LptD (Imp/OstA) and other β-barrel proteins are critical to lipid transport, and that these proteins depend on Omp85 proteins for their assembly, the lipid-based phenotypes seen in *bamA* mutants were found to be indirect effects from diminished β-barrel protein assembly. Thus, cautious acceptance of the phenotypes of loss-of-function *tam* mutants is warranted as the function of the TAM continues to be explored.

TamB is an inner membrane protein that extends through the peptidoglycan network to buttress the POTRA domains of TamA (Fig. 2), with the loss of either TamA or TamB having the same impact on TAM-mediated β-barrel protein assembly. For many β-barrel proteins of complicated topology, interaction with the TAM represents the rate-limiting step of their assembly. The increased efficiency that the TAM provides is not needed for cell viability in lab monocultures, but enables more rapid deployment of phenotypes which may matter in the context of bacteria colonizing new environmental niches, including infection sites. An intriguing prospect is that this TAM-mediated efficiency could provide a selective advantage in realizing the potential of new genes acquired through lateral gene transfer, a process that has shaped the evolution of *E. coli* and other bacterial species. It would be exciting if the TAM also proves to be directly functioning in lipid equilibration, as it would join the ranks of the relatively few protein complexes with dual biochemical activities.
